# Prevalence and factors associated with dyslipidemia in children aged 6 to 42 months in a Brazilian capital

**DOI:** 10.1590/0102-311XEN202123

**Published:** 2024-09-23

**Authors:** Vanessa Roriz Ferreira de Abreu, Lina Monteiro de Castro Lobo, Raquel Machado Schincaglia, Paulo Sérgio Sucasas da Costa, Lana Angélica Braudes-Silva, Maria Claret Costa Monteiro Hadler

**Affiliations:** 1 Escola de Ciências Sociais e da Saúde, Pontifícia Universidade Católica de Goiás, Goiânia, Brasil.; 2 Secretaria Municipal de Saúde, Goiânia, Brasil.; 3 Programa de Pós-graduação em Ciências da Saúde, Universidade Federal de Goiás, Goiânia, Brasil.; 4 Universidade Federal do Rio de Janeiro, Rio de Janeiro, Brasil.; 5 University of Nevada Las Vegas, Las Vegas, U.S.A.; 6 Programa de Pós-graduação em Nutrição e Saúde, Universidade Federal de Goiás, Goiânia, Brasil.

**Keywords:** Dyslipidemias, Hypercholesterolemia, Child Nutrition, Cholesterol, BMI-Age, Dislipidemias, Hipercolesterolemia, Nutrição da Criança, Colesterol, IMC-Idade, Dislipidemias, Hipercolesterolemia, Nutrición del Niño, Colesterol, IMC-Edad

## Abstract

This study aimed to assess the prevalence and factors associated with lipid profile abnormalities of children aged 6 to 42 months in a Central-West Brazilian capital city. This cross-sectional study used data from the baseline of a cluster-randomized clinical trial conducted in parallel. It evaluated the lipid profile, usual nutrients intake (direct food-weighing method and 24-hour dietary recall), anthropometric parameters, and socioeconomic aspects of 169 children from early childhood education centers. Poisson regression with robust variance analysis was conducted. Of the total sample, 85% had dyslipidemia, 72% had high-density lipoproteins (HDL-c) levels below the desired range, 49% had increased triglycerides (TG), 17% exhibited elevated low-density lipoproteins (LDL-c), and 15% showed high total cholesterol (TC). An increase in the body mass index (BMI) for age z-score was associated with a higher prevalence of increased TG (PR = 1.22; 95%CI: 1.05-1.41; p = 0.009). Higher age in children was associated with an increased prevalence of high LDL-c (PR = 1.037; 95%CI: 1.01-1.07; p = 0.022) and TC (PR = 1.036; 95%CI: 1.00-1.07; p = 0.037), however it was a protective factor against low HDL-c (PR = 0.991; 95%CI: 0.98-1.00; p = 0.042). High energy intake was associated with low HDL-c (PR = 1.001; 95%CI: 1.00-1.00; p = 0.023). A higher prevalence of increased LDL-c (PR = 1.005; 95%CI: 1.00-1.01; p = 0.006) and decreased HDL-c (PR = 1.002; 95%CI: 1.00-1.00; p < 0.001) were associated with dietary cholesterol intake. Most of the children presented at least one alteration in serum lipids. Lipid profile abnormalities were associated with higher BMI, older age, and increased caloric and cholesterol intake.

## Abstract (pt)

O objetivo deste estudo foi avaliar a prevalência e os fatores associados às anormalidades do perfil lipídico de crianças de 6 a 42 meses de idade em uma capital do Centro-oeste do Brasil. Este estudo transversal utilizou dados da linha de base de um ensaio clínico randomizado por conglomerados realizado em paralelo. Avaliou-se o perfil lipídico, a ingestão habitual de nutrientes (método de pesagem direta de alimentos e recordatório alimentar de 24 horas), parâmetros antropométricos e aspectos socioeconômicos de 169 crianças de centros de educação infantil. Foi realizada uma regressão de Poisson com análise de variância robusta. Da amostra total, 85% apresentavam dislipidemia, 72% tinham níveis de lipoproteínas de alta densidade (HDL-c) abaixo da faixa desejada, 49% tinham triglicerídeos (TG) aumentados, 17% apresentavam lipoproteínas de baixa densidade (LDL-c) elevadas e 15% apresentavam colesterol total (CT) elevado. Um aumento no índice de massa corporal (IMC) para o escore z da idade foi associado a uma maior prevalência de aumento de TG (RP = 1,22; IC95%: 1,05-1,41; p = 0,009). A idade mais avançada das crianças foi associada a uma maior prevalência de LDL-c alto (RP = 1,037; IC95%: 1,01-1,07; p = 0,022) e CT (RP = 1,036; IC95%: 1,00-1,07; p = 0,037), mas foi um fator de proteção contra HDL-c baixo (RP = 0,991; IC95%: 0,98-1,00; p = 0,042). A alta ingestão de energia foi associada ao baixo HDL-c (RP = 1,001; IC95%: 1,00-1,00; p = 0,023). Uma maior prevalência de aumento de LDL-c (RP = 1,005; IC95%: 1,00-1,01; p = 0,006) e diminuição de HDL-c (RP = 1,002; IC95%: 1,00-1,00; p < 0,001) foi associada à ingestão de colesterol na dieta. A maioria das crianças apresentou pelo menos uma alteração nos lipídios séricos. As anormalidades do perfil lipídico foram associadas ao IMC mais alto, à idade mais avançada e ao aumento da ingestão calórica e de colesterol.

## Abstract (es)

El objetivo de este estudio fue evaluar la prevalencia y los factores asociados con las anomalías del perfil lipídico en niños de 6 a 42 meses de edad en una ciudad del Centro-Oeste de Brasil. Este estudio transversal utilizó datos de referencia de un ensayo clínico aleatorizado por grupos realizado en paralelo. Se evaluó el perfil lipídico, la ingesta habitual de nutrientes (pesaje directo de alimentos y recordatorio de alimentación de 24 horas), los parámetros antropométricos y los aspectos socioeconómicos de 169 niños de centros de educación infantil. Se realizó una regresión de Poisson con un análisis robusto de la varianza. Se reveló que el 85% de los participantes tenían dislipidemia; el 72% presentaron niveles de lipoproteínas de alta densidad (HDL-c) por debajo del rango esperado; el 49% tenía aumento de triglicéridos (TG); el 17% de lipoproteínas de baja densidad (LDL-c) elevadas y el 15% tenía colesterol total (CT) elevado. Un aumento en el índice de masa corporal (IMC) para la puntuación z de la edad se asoció con una mayor tendencia a aumento de TG (RP = 1,22; IC95%: 1,05-1,41; p = 0,009). La edad más avanzada de los niños se asoció con una mayor prevalencia de LDL-c alta (RP = 1,037; IC95%: 1,01-1,07; p = 0,022) y CT (RP = 1,036; IC95%: 1,00-1,07; p = 0,037), pero fue un factor de protección contra HDL-c baja (RP = 0,991; IC95%: 0,98-1,00; p = 0,042). La ingesta alta de energía se asoció con HDL-c baja (RP = 1,001; IC95%: 1,00-1,00; p = 0,023). Se encontraron que la mayor tendencia a aumento de LDL-c (RP = 1,005; IC95%: 1,00-1,01; p = 0,006) y disminución de HDL-c (RP = 1,002; IC95%: 1,00-1,00; p < 0,001) estuvieron asociadas con la ingesta de colesterol en la dieta. La mayoría de los niños tuvieron al menos un cambio en los lípidos séricos. Las anomalías del perfil lipídico se asociaron con un mayor IMC, mayor edad y mayor consumo de calorías y colesterol.

## Introduction

The rising prevalence of dyslipidemia in children emerges as a notable concern at childhood, evidenced by alterations in the levels of blood lipoproteins, including triglycerides (TG), cholesterol, and high- and low-density lipoproteins (HDL-c and LDL-c, respectively). The main causes of dyslipidemia in childhood are unhealthy lifestyle habits, including diets rich in saturated or trans fats, and physical inactivity. Dyslipidemias can be classified, according to the altered lipid fraction, into the following categories: isolated hypercholesterolemia (increased LDL-c), isolated hypertriglyceridemia (increased TG), mixed hyperlipidemia (increased LDL-c and TG), and low HDL-c (either isolated HDL-c reduction or in association with an increase in LDL-c or TG) ^1^.

These changes in lipid fractions play a central role in the development of atherosclerotic cardiovascular diseases ^2^. Clinical manifestations of the atherothrombotic events are more frequent in adulthood. However, early exposure to a hyperlipidemia environment can lead to lipid deposition in the artery walls as early as the first weeks after conception ^3^. Atherosclerosis can begin in childhood, and serum lipoproteins levels found at this stage tend to remain stable throughout life ^4^ ^,^ ^5^. Fatty streaks can be observed in the intimal layer of the aorta as early as the age of three, and they may progress during the third and fourth decades of life ^6^.

Brazilian population studies report prevalence of elevated LDL-c in children ranging from 10% to 47% ^7^ ^,^ ^8^ ^,^ ^9^ ^,^ ^10^ ^,^ ^11^. These data highlight the need for timely diagnostic, as well as therapeutic and preventive measures, considering that childhood is a strategic phase in the prevention of atherosclerosis at the population level for the formation of lifestyle habits, which are important causes of cardiovascular risk modulation ^12^.

The primary determinants of dyslipidemias are excess weight, diet, physical activity, and genetics. Socioeconomic vulnerability, particularly factors such as low maternal education, and early weaning, is also associated with elevated blood lipids ^11^ ^,^ ^13^. Regarding diet, it is known that increases in total cholesterol (TC) and TG concentrations can be caused by increased intake of cholesterol, carbohydrates, saturated fats, and trans fatty acids. Additionally, increased energy is associated with these changes ^14^. Data from the *Brazilian National Health Survey* (PNS, acronym in Portuguese) show high prevalences of consumption of soft drinks, sweets, biscuits, porridge, and other ultra-processed foods, which are high in calories and rich in fats, among children under 2 years of age ^15^.

Screening children’s lipid profiles is important to assist health promotion policies aimed at reducing cardiovascular risk factors in this population. Furthermore, identifying risk factors associated with dyslipidemia in childhood can contribute to the control of this pathology and reduction of cardiovascular mortality ^16^. This study was conducted due to the significance of this subject, the high prevalence of dyslipidemia in childhood, and the lack of research in this age group. Therefore, this study aimed to evaluate the prevalence and factors associated with lipid profile abnormalities in children aged 6 to 42 months in a Brazilian capital city.

## Materials and methods

This is a cross-sectional study that used a baseline data from a parallel cluster-randomized clinical trial titled *Effect of Fortification with Multiple Micronutrient Powder on the Prevention and Treatment of Iron Deficiency and Anaemia in Brazilian Children: A Randomized Clinical Trial*, carried out by the Faculty of Nutrition at the Federal University of Goiás (UFG, acronym in Portuguese), Brazil. The matrix study was previously described in details by Machado et al. ^17^.

The sample consisted of children aged between 6 and 42 months, from early childhood education centers (CMEIs, acronym in Portuguese) in the city of Goiânia (Goiás State), located in the Central-West region of Brazil. The age range of the NutriSUS Strategy - Brazilian Ministry of Health is from 6 to 48 months, therefore, the children were selected at baseline with the criteria of being from 6 to 42 months of age, for them to correspond the age range after the intervention. CMEIs are public institutions linked to the Goiânia Municipal Department of Education. Professionals trained in pedagogy take care of the children for up to eight hours a day, in this first stage of Brazilian primary education (from 6 months to 5 years and 11 months). The exclusion criteria was premature children, twins, those with low birth weight, as well as children being treated for anemia, malaria, HIV or haemoglobinopathies. Children allergic to any of the offered micronutrients were also excluded. Additionally, children without available results of the lipid profile test were excluded.

In the city, CMEIs that had already received fortification with multiple micronutrient powders, those lacking a nursery, or those not operating full-time were excluded from the study. Of the seven existing health districts in Goiânia, five were randomly selected and, subsequently, two CMEIs were randomly selected per health district. In a subsequent random draw, for each district one group was selected to receive ferrous sulfate heptahydrate plus folic acid, while the other received NutriSUS. The selection was performed using a random number list generated by Epi Info 6.04d (https://www.cdc.gov/epiinfo/index.html). By employing a simple random draw function in Excel 365 (https://products.office.com/), the randomization process was stratified by gender and age group ^17^.

Collection took place between March 2018 and March 2019, in the city of Goiânia, after the parents or children’ guardians signed the Informed Consent Form. Interviews were conducted in CMEIs by trained undergraduate students of Nutrition, and supervised by a nutritionist, or master and doctoral students in Nutrition. The registered variables were: child age (months), sex (male/female), race or skin color (white vs. non-white), maternal age (years), maternal education (completed years), monthly income per capita (converted from BRL 3.60 to USD 1.00 at the exchange rate on May 9, 2018), breastfeeding maternal status (current practice - yes/no), duration of exclusive breastfeeding (days), body mass index for age (BMI-for-age z-score) and height for age (z-score) ^18^.

Weight and height measurements were taken in duplicate at the CMEIs. For the body weight measurement, a SECA 877 digital scale (https://www.seca.com) with a capacity of 200kg and an accuracy of 100g was used. For height measurement, the child was placed in an orthostatic position using a portable stadiometer (Alturexata; https://alturexata.com.br/) affixed to a baseboard-free wall. For children under 2 years of age, length was assessed in the supine position.

Blood collection was performed by a technician from the laboratory responsible for the analyses. Children who did not attend the CMEI on the date of the blood collection had their samples collected at home or at the laboratory. Venous blood was collected after a fasting period of three hours (for children under 12 months) to eight hours. TC, HDL-c, and TG were determined by the enzymatic colorimetric method, with automation by the Cobas 8000 c502 equipment (Roche Corporation; https://www.roche.com/), following the manufacturer’s recommendations. LDL-c concentrations were calculated using the Friedewald formula ^19^.

Additionally, the following lipid profile variables were determined for individuals older than 2 years: increased TC (≥ 170mg/dL), decreased HDL-c (≤ 45mg/dL), increased TG (≥ 75mg/dL), increased LDL-c (≥ 110mg/dL) ^1^ ^,^ ^20^ ^,^ ^21^, and alteration in at least one of the previously mentioned lipid profile parameters (at least one altered parameter). For children aged 6 to 23 months, reference values from the U.S. National Cholesterol Education Program (NCEP) - Expert Panel on Blood Cholesterol Levels in Children and Adolescents ^22^ were used, due to the absence of specific recommendations for this age group within Brazilian guidelines ^1^. Lipoprotein concentrations thresholds for children aged 6 to 23 months are defined as follows: high TC (≥ 200mg/dL), high TG (≥ 100mg/dL), high LDL-c (≥ 130mg/dL), and low HDL-c (< 40mg/dL) ^22^.

Dietary intake for one day was assessed, and for two days in 20% of the sample (one month interval), by direct food-weighing and 24-hour dietary recall (24hR). Weighing was carried for all meals served at the CMEI (breakfast, morning snack, lunch, afternoon snack, and dinner), using a BEL precision balance, model S6501 (https://www.belengineering.com), with an accuracy of 0.01g, according to the procedures by Cruz et al. ^23^. The 24hR was applied to the parents or guardians of the child on the same day as the direct food-weighing, for nutrients consumption assessment outside of the CMEI, to obtain information about the child’s complete eating day, with the aid of utensils and photographic records of foods, following the multiple-pass method (MPM) ^24^. According to specific tables, all preparations mentioned in household measurements were converted into grams or milliliters ^25^ ^,^ ^26^ ^,^ ^27^. Nutrients intake was assessed by the sum of data from direct food: weighing + 24hR.

Total energy intake and the following nutrients were assessed: energy (kcal), carbohydrates (g), protein (g), total fat (g), saturated fat (g), trans fatty acids (g), monounsaturated fats (g), polyunsaturated fats (g), omega-6 (g), omega-3 (g), omega-6 to omega-3 ratio (ω-6/ω-3 ratio), cholesterol (mg), and dietary fiber (g). Dietary intake was analyzed using the dietWin Profissional Plus software (dbv3090; http://www.dietwin.com.br). Data were double-entered into Excel version 365 and validated in Epi-Info 6.04d. The statistical modeling software, Multiple Source Method (MSM; https://msm.dife.de/), was used to estimate usual macronutrients and energy intake, reducing intra-individual variance ^28^.

The database was created using Excel for Windows (version 10), with double entry to check data consistency. Normality was assessed using the Shapiro-Wilk test (p > 0.05). The sample was characterized by absolute and relative frequencies for the categorical variables, mean and standard deviation for the parametric variables, and median and interquartile range (IQR) for the nonparametric variables. A boxplot was constructed for better visualization of nonparametric data. Pearson’s chi-square test or Fisher’s exact test were used to assess the association between the independent categorical variables and the outcome. The Mann-Whitney U-test and t-test were applied to compare the independent numeric variables with the outcome (lipid profile). Poisson regression with robust variance analysis was conducted, estimating the prevalence ratio (PR) with a 95% confidence interval (95%CI) ^29^. Variables with p < 0.20 were selected for the multiple regression model and adjusted pseudo R-squared (pseudo R^2^), estimated using the same regression statistical method. A significance level of 5% was adopted for all tests. Analyses were performed using Stata software, version 16.0 (https://www.stata.com).

The study received approval from the Research Ethics Committee of the UFG (protocol n. 3,692,768). It was also registered with the Brazilian Registry of Clinical Trials (REBEC, acronym in Portuguese; protocol RBR-4hm7mz). The research strictly adhered to the criteria set by *Resolution n. 466/2012* of the Brazilian National Health Council concerning research standards involving human subjects.

## Results

Out of the initial 205 children who began the study, 169 were included in the present analysis, with the remaining participants being filtered based on inclusion and exclusion criteria [excluded (n = 36): not meeting inclusion criteria (n = 16), declined to participate (n = 4), without lipid profile test (n = 15), and other reasons (n = 1)]. The sample characteristics are detailed in Table 1. Slightly over 50% were girls and identified as belonging to a non-white racial or ethnic group (50% mixed race and 5% black). Breastfeeding is still practiced to feed approximately one-third of the children, with the median duration of exclusive breastfeeding being 4 months. Details on children’s nutrient intake are provided in Table 1.

**Table 1:** Socioeconomic, demographic, anthropometric, and nutritional consumption characterization of the total sample and by dyslipidemia in children of 6 to 42 months of age. Goiânia, Goiás State, Brazil, 2018-2019.

Characteristics	Sample	At least one altered parameter	Increased TG	Increased TC	Decreased HDL-c	Increased LDL-c
n (%)	169 (100)	144 (85)	83 (49)	26 (15)	122(72)	29 (17)
Child age (months) [median (IQR]	24 (15-35)	24 (13-35)	24 (13-34)	30 (24-34)	24 (13-34)	31 (24-35)
p-value		0.720	0.059	0.088	0.119	0.032
Sex [n (%)]						
Female	89 (53)	74 (51)	43 (52)	14 (54)	63 (52)	14 (48)
Male	80 (47)	70 (49)	40 (48)	12 (46)	62 (48)	15 (52)
p-value		0.426 *	0.827 *	0.895 *	0.668 *	0.603 *
Race or skin color [n (%)]						
White	76 (45)	62 (43)	35 (42)	10 (38)	52 (43)	10 (34)
Non-white	93 (55)	82 (57)	48 (58)	16 (62)	70 (57)	19 (66)
p-value		0.230 *	0.472 *	0.468 *	0.323 *	0.212 *
Maternal age (years) [mean (SD)]	30 (6)	30 (6)	30 (6)	30 (6)	30 (6)	30 (4)
p-value		0.106 **	0.827 **	0.853 **	0.281 **	0.934 **
Maternal education (years) [median (IQR)]	12 (12-16)	12 (12-16)	12 (12-16)	12 (11-16)	12 (12-15)	12 (11-16)
p-value		0.902	0.569	0.731	0.990	0.821
Monthly income per capita (USD) ^#^ [median (IQR)]	206 (132-278)	204 (131-278)	208 (125-278)	208 (139-312)	206 (132-278)	208 (139-278)
p-value		0.860	0.864	0.727	0.774	0.702
Current breastfeeding practice [n (%)]	45 (31)	41 (32)	23 (32)	9 (39)	31 (29)	8 (32)
p-value		0.429 ***	0.815 *	0.334 *	0.403 *	0.869 *
Duration of exclusive breastfeeding (days) [median (IQR)]	122 (61-183)	122 (45-182)	122 (61-152)	122 (61-182)	106 (61-182)	122 (61-182)
p-value		0.052	0.279	0.998	0.195	0.420
BMI-for-age (z-score) [mean (SD)]	0.8 (1.0)	0.8 (1.0)	1.0 (1.1)	0.8 (1.1)	0.8 (1.1)	0.8 (1.1)
p-value		0.917 **	0.007 **	0.807 **	0.513 **	0.882 **
Height (z-score) [median (IQR)]	-0.2 (-0.9-0.8)	-0.2 (-0.9-0.8)	-0.2 (-0.9-0.7)	-0.2 (-1.0-0.5)	-0.2 (-0.9-0.8)	-0.1 (-0.1-0.6)
p-value		0.280	0.454	0.672	0.431	0.920
Energy (kcal) [median (IQR)]	1,256 (1,149-1,353)	1,258 (1,146-1,358)	1,250 (1,133-1,355)	1,254 (1,151-1,377)	1,256 (1,143-1,362)	1,257 (1,146-1,377)
p-value		0.769	0.667	0.956	0.967	0.956
Carbohydrates (g) [mean (SD)]	179 (14)	179 (15)	177 (15)	176 (15)	179 (14)	176 (15)
p-value		0.804 **	0.346 **	0.348 **	0.918 **	0.317 **
Protein (g) [median (IQR)]	48 (39-58)	49 (39-59)	49 (39-59)	50 (40-62)	48 (38-59)	52 (37-62)
p-value		0.489	0.877	0.622	0.643	0.425
Fat (g) [mean (SD)]	39 (6)	38 (6)	38 (6)	38 (6)	38 (6)	38 (6)
p-value		0.411 **	0.529 **	0.382 **	0.274 **	0.411 **
Saturated fat (g) [median (IQR)]	14 (11-17)	14 (11-17)	15 (12-17)	14 (12-17)	14 (11-17)	14 (11-17)
p-value		0.154	0.986	0.716	0.070	0.610
Trans fatty acids (g) [median (IQR)]	1.0 (0.6-1.5)	0.9 (0.6-1.5)	0.9 (0.6-1.4)	1.1 (0.7-1.3)	0.9 (0.6-1.5)	1.1 (0.7-1.4)
p-value		0.087	0.050	0.978	0.063	0.914
Monounsaturated fat (g) [median (IQR)]	9 (8-12)	10 (8-12)	9 (7-12)	10 (8-12)	10 (7-12)	10 (8-13)
p-value		0.472	0.164	0.879	0.203	0.713
Polyunsaturated fat (g) [median (IQR)]	6 (5-7)	6 (5-7)	6 (5-7)	6 (5-7)	6 (5-7)	6 (5-8)
p-value		0.828	0.336	0.699	0.487	0.961
Omega-6 (g) [median (IQR)]	5 (4-6)	5 (4-6)	5 (4-6)	4 (4-6)	5 (4-6)	5 (4-6)
p-value		0.885	0.259	0.203	0.533	0.318
Omega-3 (g) [median (IQR)]	0.8 (0.6-0.9)	0.8 (0.6-0.9)	0.8 (0.6-0.9)	0.8 (0.5-0.9)	0.8 (0.6-0.9)	0.8 (0.5-1.1)
p-value		0.524	0.456	0.725	0.212	0.915
ω-6/ω-3 ratio [median (IQR)]	6 (6-7)	7 (6-8)	7 (6-8)	6 (6-7)	7 (6-8)	7 (6-7)
p-value		0.539	0.870	0.569	0.459	0.795
Cholesterol (mg) [median (IQR)]	131 (100-180)	132 (102-182)	134 (103-185)	132 (101-204)	132 (105-181)	137 (120-209)
p-value		0.409	0.367	0.569	0.318	0.162
Fiber total (g) [median (IQR)]	11 (8-13)	11 (9-14)	11 (9-13)	11 (9-12)	12 (9-14)	11 (9-13)
p-value		0.107	0.438	0.257	0.144	0.474

BMI: body mass index; IQR: interquartile range; HDL-c: high-density lipoproteins; LDL-c: low-density lipoproteins; SD: standard deviation; TC: total cholesterol; TG: triglycerides.

Note: p-value obtained using the Mann-Whitney U test, except for: * Pearson’s chi-square test; ** t-test; *** Fisher’s exact test. Bold values: p < 0.05.

^#^ Converted form BRL 3.60 to USD 1.00.

In examining the associations between dyslipidemia diagnoses and sociodemographic, maternal, breastfeeding-related, anthropometric, and nutritional consumption variables, it was noted that children with increased triglycerides exhibited higher BMI-for-age z-score, and individuals with increased LDL-c levels were older (Table 1). The PRs of associations between socioeconomic, demographic, anthropometric, and nutritional consumption factors with dyslipidemia were presented using simple Poisson regression with robust variance adjustment model in Table 2, and with a multiple Poisson regression model as outlined in Table 3.

**Table 2:** Association between socioeconomic, demographic, anthropometric, and nutritional consumption with dyslipidemia in children of 6 to 42 months of age. Goiânia, Goiás State, Brazil, 2018-2019.

Characteristics	At least one altered parameter	Increased TC	Decreased HDL-c	Increased LDL-c	Increased TG
	PR (95%CI)	PR (95%CI)	PR (95%CI)	PR (95%CI)	PR (95%CI)
Child age (months)	1.00 (0.99-1.00)	1.03 (1.00-1.07)	0.99 (0.98-1.00)	1.04 (1.01-1.07)	1.00 (0.99-1.01)
p-value	0.694	0.047	0.093	0.017	0.572
Sex	1.05 (0.93-1.19)	0.95 (0.47-1.94)	1.04 (0.86-1.26)	1.19 (0.61-2.32)	1.03 (0.76-1.41)
p-value	0.425	0.896	0.668	0.605	0.827
Race or skin color	1.08 (0.95-1.23)	1.31 (0.63-2.72)	1.10 (0.91-1.33)	1.55 (0.77-3.14)	1.12 (0.82-1.53)
p-value	0.243	0.473	0.332	0.221	0.477
Maternal age (years)	0.99 (0.98-1.00)	1.01 (0.95-1.07)	0.99 (0.97-1.01)	1.00 (0.96-1.05)	1.00 (0.97-1.02)
p-value	0.162	0.844	0.318	0.913	0.826
Maternal education (years)	1.01 (0.98-1.03)	1.00 (0.89-1.12)	1.00 (0.97-1.04)	1.00 (0.90-1.11)	1.02 (0.97-1.08)
p-value	0.640	0.971	0.864	0.969	0.449
Monthly income per capita (USD)	1.00 (1.00-1.00)	1.00 (1.00-1.00)	1.00 (1.00-1.00)	1.00 (1.00-1.00)	1.00 (1.00-1.00)
p-value	0.306	0.305	0.445	0.556	0.753
Current breastfeeding practice	0.94 (0.83-1.06)	0.69 (0.32-1.47)	1.10 (0.87-1.37)	0.94 (0.44-2.02)	0.96 (0.68-1.36)
p-value	0.290	0.333	0.428	0.869	0.814
Duration of exclusive breastfeeding (days)	1.00 (1.00-1.00)	1.00 (0.99-1.01)	1.00 (1.00-1.00)	1.00 (1.00-1.01)	1.00 (1.00-1.00)
p-value	0.063	0.904	0.225	0.339	0.370
BMI-for-age (z-score)	1.00 (0.94-1.06)	1.04 (0.73-1.48)	1.03 (0.94-1.12)	0.98 (0.70-1.36)	1.22 (1.05-1.41)
p-value	0.922	0.814	0.503	0.887	0.009
Height-for-age (z-score)	0.96 (0.92-1.00)	0.95 (0.73-1.25)	0.97 (0.90-1.05)	1.03 (0.78-1.36)	0.93 (0.82-1.05)
p-value	0.110	0.732	0.419	0.832	0.216
Energy (kcal)	1.00 (1.00-1.00)	1.00 (1.00-1.00)	1.00 (1.00-1.00)	1.00 (1.00-1.00)	1.00 (1.00-1.00)
p-value	0.695	0.705	0.863	0.719	0.574
Carbohydrates (g)	1.00 (1.00-1.00)	0.99 (0.96-1.01)	1.00 (0.99-1.01)	0.99 (0.96-1.01)	0.99 (0.98-1.01)
p-value	0.768	0.357	0.918	0.336	0.351
Protein (g)	1.00 (1.00-1.00)	1.00 (0.98-1.02)	1.00 (1.00-1.01)	1.01 (0.99-1.03)	1.00 (0.99-1.01)
p-value	0.421	0.821	0.369	0.526	0.872
Fat (g)	1.00 (0.99-1.00)	0.97 (0.91-1.04)	0.99 (0.98-1.01)	0.98 (0.92-1.04)	0.99 (0.96-1.02)
p-value	0.337	0.383	0.278	0.439	0.531
Saturated fat (g)	0.99 (0.98-1.00)	0.97 (0.89-1.05)	0.98 (0.96-1.01)	0.97 (0.89-1.05)	1.00 (0.97-1.04)
p-value	0.205	0.462	0.146	0.472	0.890
Trans fatty acids (g)	0.95 (0.88-1.04)	0.95 (0.60-1.49)	0.90 (0.77-1.04)	0.98 (0.65-1.49)	0.84 (0.65-1.10)
p-value	0.252	0.824	0.155	0.938	0.202
Monounsaturated fat (g)	0.99 (0.98-1.01)	1.01 (0.90-1.13)	0.98 (0.95-1.01)	1.02 (0.91-1.13)	0.97 (0.93-1.02)
p-value	0.537	0.919	0.149	0.750	0.295
Polyunsaturated fat (g)	1.00 (0.97-1.03)	0.98 (0.77-1.25)	0.98 (0.93-1.04)	1.02 (0.82-1.27)	0.95 (0.87-1.04)
p-value	0.952	0.875	0.481	0.863	0.302
Omega-6 (g)	1.00 (0.97-1.03)	0.90 (0.67-1.21)	0.98 (0.92-1.04)	0.95 (0.73-1.22)	0.94 (0.85-1.04)
p-value	0.992	0.491	0.461	0.671	0.233
Omega-3 (g)	0.97 (0.82-1.16)	0.57 (0.14-2.32)	0.86 (0.62-1.21)	0.76 (0.20-2.90)	0.76 (0.42-1.36)
p-value	0.770	0.435	0.388	0.686	0.356
ω-6/ω-3 ratio	1.02 (0.99-1.04)	0.93 (0.74-1.17)	1.03 (0.99-1.07)	0.95 (0.77-1.17)	0.96 (0.86-1.07)
p-value	0.290	0.546	0.179	0.616	0.468
Cholesterol (mg)	1.00 (1.00-1.00)	1.00 (1.00-1.01)	1.00 (1.00-1.00)	1.00 (1.00-1.01)	1.00 (1.00-1.00)
p-value	0.156	0.282	0.081	0.033	0.222
Fiber total (g)	1.01 (0.99-1.03)	0.93 (0.85-1.02)	1.01 (0.98-1.04)	0.97 (0.88-1.06)	0.98 (0.94-1.03)
p-value	0.239	0.111	0.561	0.473	0.481

95%CI: 95% confidence interval; BMI: body mass index; HDL-c: high-density lipoproteins; LDL-c: low-density lipoproteins; PR: prevalence ratio; TC: total cholesterol; TG: triglycerides.

Note: simple Poisson’s regression. Bold values: considered in the multiple model (p < 0.20).

**Table 3:** Association between the socioeconomic, demographic, anthropometric, and nutritional consumption and dyslipidemia in children of 6 to 42 months of age. Goiânia, Goiás State, Brazil, 2018-2019.

Characteristics	At least one altered parameter *		Increased TC **		Decreased HDL-c ***		Increased LDL-c ^#^	
	PR (95%CI)	p-value	PR (95%CI)	p-value	PR (95%CI)	p-value	PR (95%CI)	p-value
Child age (months)			1.036 (1.00-1.07)	0.037	0.991 (0.98-1.00)	0.042	1.037 (1.01-1.07)	0.022
Maternal age (years)	0.993 (0.98-1.01)	0.278						
Duration of exclusive breastfeeding (days)	0.999 (1.00-1.00)	0.059						
Height-for-age (z-score)	0.970 (0.93-1.01)	0.184						
Energy (kcal)	1.000 (1.00-1.00)	0.928	1.000 (1.00-1.00)	0.757	1.001 (1.00-1.00)	0.023	0.998 (1.00-1.00)	0.121
Saturated fat (g)					0.974 (0.94-1.00)	0.090		
Trans fatty acids (g)					0.954 (0.80-1.14)	0.598		
Monounsaturated fat (g)					0.973 (0.93-1.02)	0.274		
ω-6/ω-3 ratio					1.023 (0.97-1.08)	0.393		
Cholesterol (mg)	1.001 (1.00-1.00)	0.069			1.002 (1.00-1.00)	< 0.001	1.005 (1.00-1.01)	0.006
Fiber total (g)			0.897 (0.79-1.02)	0.096				

95%CI: 95% confidence interval; HDL-c: high-density lipoproteins; LDL-c: low-density lipoproteins; PR: prevalence ratio; TC: total cholesterol; TG: triglycerides.

Note: adjusted multiple Poisson regression models with robust variance. Covariate selection based on variables with p < 0.20 in binary regression analysis. Bold values indicate statistical significance (p < 0.05).

* Pseudo R^2^ = 0.0034 (n = 153);

** Pseudo R^2^ = 0.0333 (n = 160);

*** Pseudo R^2^ = 0.0137 (n = 160);

^#^ Pseudo R^2^ = 0.0450 (n = 160).

Older age was associated with a higher prevalence of children having increased TC and LDL-c. On the other hand, older age was associated with a decreased prevalence of reduced HDL-c (Table 3). Additionally, an increased intake of dietary cholesterol and energy was associated with a higher prevalence of reduced HDL-c levels in children. A higher prevalence of increased LDL-c was also associated with higher dietary cholesterol intake (Table 3). Finally, a higher BMI z-score was associated with a higher prevalence of elevated TG (Table 2).

**Figure 1:**
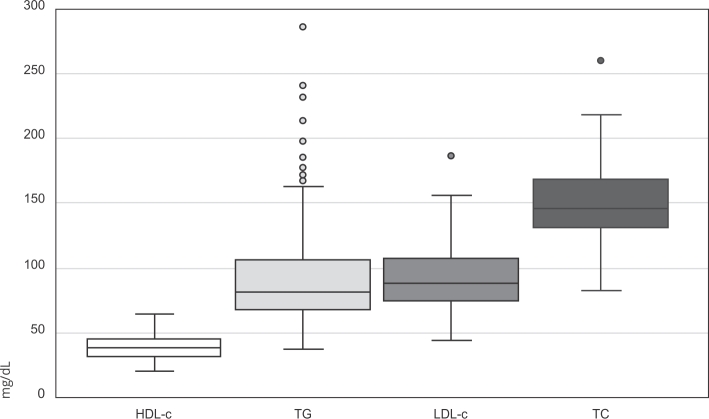
Box plot representation of lipid profile fractions in children of 6 to 42 months of age. Goiânia, Goiás State, Brazil, 2018-2019.

**Figure 2:**
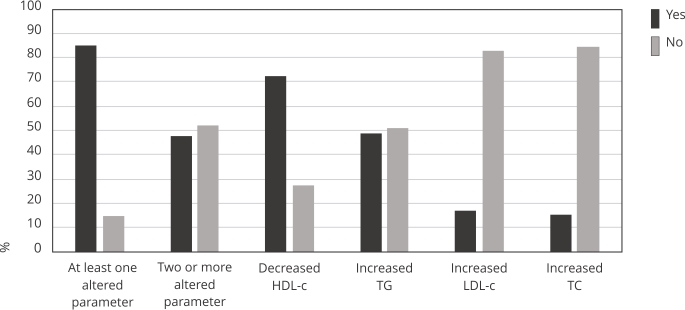
Prevalence of dyslipidemia diagnoses in children of 6 to 42 months of age. Goiânia, Goiás State, Brazil, 2018-2019.

The median TC was 146mg/dL (IQR: 131-169), HDL-c was 38mg/dL (IQR: 32-45), LDL-c was 88mg/dL (IQR: 74-107), and TG was 81mg/dL (IQR: 68-105) (Figure 1). It was determined that 48% of the sample population had at least two altered lipid parameters. In descending order of frequency, these were: low HDL-c, high TG, high LDL-c, and elevated TC (Figure 2). Regarding children older than 2 years, the prevalences of low HDL-c, high TG, high LDL-c, and elevated TC were 72%, 54%, 27%, and 24%, respectively.

HDL-c: high-density lipoproteins; LDL-c: low-density lipoproteins; TC: total cholesterol; TG: triglycerides.

HDL-c: high-density lipoproteins; LDL-c: low-density lipoproteins; TC: total cholesterol; TG: triglycerides.

## Discussion

This study highlighted a significant finding: high prevalence of dyslipidemia in the sample, primarily characterized by reduced HDL-c and elevated TG. Data analysis showed that changes in the lipid profile were associated with age (older age linked to increased TC and LDL-c, and younger age linked to lower HDL-c), higher BMI-for-age (z-score), and increased dietary intake of calories and cholesterol. Notably, serum lipid levels did not display significant differences across genders, races, maternal age and education levels, income, breastfeeding practices, height, and certain dietary variables (such as carbohydrates, protein, total fat, saturated fat, trans fatty acids, monounsaturated fat, polyunsaturated fat, omega-6, omega-3, ω-6/ω-3 ratio, and fiber).

The proportion of children with low HDL-c found in this research exceeds some studies, which reported frequencies of 54% ^9^ and 57% ^8^ in the Brazilian states Ceará and Rio Grande do Sul, respectively. The observed frequency of high TC in our study is lower than that reported in other national studies, which have documented rates between 33% and 45% ^8^ ^,^ ^9^ ^,^ ^10^ ^,^ ^11^. For instance, one study conducted with 700 children aged 2 to 9 years in a Northeastern region also identified a high prevalence of elevated TC (45%) and dyslipidemia (68%) ^9^. In another study by Gomes et al. ^10^, 67% of the lipid profiles indicated the presence of at least one type of dyslipidemia.

Regardless of the cutoff points used for the diagnosis or the techniques applied in biochemical analysis, it is significant to emphasize that the prevalence of increased TG, but not high TC, observed in our study is higher than what has been reported in other Brazilian studies ^7^ ^,^ ^8^ ^,^ ^9^ ^,^ ^10^ ^,^ ^11^ ^,^ ^13^. Moreover, our findings for high LDL-c exceed those of some studies ^7^ ^,^ ^8^ ^,^ ^13^, while falling below others ^9,10,11^. This difference may be attributed to the inclusion of children older than 2 ^7^ ^,^ ^9^ or 4 ^11^ ^,^ ^13^ years in their samples. Previous research has indicated that pediatric dyslipidemia is a predictor of dyslipidemia and increased intima-media thickness of the carotid arteries in adulthood ^2^ ^,^ ^4^ ^,^ ^5^ ^,^ ^21^. LDL-c is considered the most atherogenic lipid fraction in the blood, elevating the risk of cardiovascular morbidity in later life ^8^.

High levels of TC and LDL-c were found to be associated with older age. Few studies have reported the prevalence of dyslipidemia in children under 4 years of age, primarily because of the challenges of blood collection, the requirement for higher fat intake to support myelination ^1^, and the metabolic instability observed during the rapid growth phase before 24 months of life ^10^. The concentrations of lipids and lipoproteins experience significant fluctuations throughout different stages of human growth. Typically, there are two periods marked by a pronounced increase in these levels: the first three years of life and the conclusion of puberty ^8^.

Based on this theoretical foundation, we considered different cutoff points for the classification of children under the age of 24 months. TC and LDL-C levels increase rapidly during the first weeks of life, followed by a more gradual rise until the age of 2 years ^30^. This pattern underlies the high prevalence of dyslipidemia observed in the current study. Screening for lipid disorders is, therefore, generally recommended after two years of age (when lipid and lipoprotein levels become quite constant until adolescence), if the child exhibits clinical signs (such as xanthomas or corneal arcus), risk factors (including hypertension, diabetes, or obesity), or family history of hypercholesterolemia or cardiovascular disease ^1^ ^,^ ^30^. Our lipid profile data offer new scientific insights to reevaluate this recommendation of initiating serum lipid profile screenings from the age of 2 years.

An increase in the BMI-for-age z-score was associated with a higher prevalence of hypertriglyceridemia in our study (PR = 1.22; 95%CI; 1.05-1.41). Comparable findings have been reported by other studies involving students aged 6 to 17 years from the Brazilian states of Minas Gerais ^31^, Rio Grande do Sul ^32^ ^,^ ^33^, and Rio de Janeiro ^34^. An elevated BMI-for-age z-score has been linked with increased LDL-c cholesterol levels and decreased HDL-c cholesterol levels in a cross-sectional study of 218 individuals between the ages of 2 and 18 years ^35^. Insulin resistance is recognized as the primary mechanism underlying the correlation between BMI-for-age z-score and lipid profiles ^13^.

The elevation of TG could be attributed to the accumulation of chylomicrons and/or very low-density lipoproteins (VLDL-c). This may be a result of decreased TG hydrolysis in these lipoproteins or an increase in the synthesis of VLDL-c. Changes in dietary habits, coupled with increased physical activity and reduced intake of fats and/or carbohydrates for weight control, are also recommended for the management of hypertriglyceridemia ^36^.

Regarding dietary variables, cholesterol intake was associated with increased LDL-c and decreased HDL-c levels. Similarly, a study examining the diets of 227 preschoolers revealed that frequent consumption of convenience bakery products, which do not require preparation before consumption, as well as foods high in lipids, displayed a pronounced association with disruptions in lipid profiles, particularly elevated LDL-c levels ^37^. From this standpoint, the Brazilian guidelines on dyslipidemia emphasize the advantages of eliminating trans fats, controlling intake of saturated fats and cholesterol, favoring polyunsaturated (especially omega-3) and monounsaturated fats, and limiting sugar consumption ^1^ ^,^ ^38^.

Contemporary research points to an imbalance in the Western dietary pattern, wherein excessive omega-6 polyunsaturated fatty acids and a disproportionately high ω-6/ω-3 ratio may drive the onset of numerous health conditions, including dyslipidemia ^39^. It is worth noting that vegetable oils are primary sources of omega-6 fatty acids, especially those from sunflower, corn, and soy, which are components of a large portion of ultra-processed foods ^27^ ^,^ ^40^. However, the ω-6/ω-3 ratio was not associated with the lipid profile in this study.

Another noteworthy finding of this study is the association of energy intake and higher prevalence of low HDL-c. This observation is significant as it underscores the urgency to assess the quality of CMEIs menus as well as meals provided at home, especially during weekends, when caregivers tend to be more lenient regarding the energy content of children’s diets. Currently, the prevailing perspective suggests that the consumption of ultra-processed foods, dining out, and replacing traditional meals with snacks contribute to excessive calorie ^7^ ^,^ ^40^. The latest *Brazilian Household Budget Survey* (POF 2017/2018) emphasized the increase of ultra-processed products in household diets, constituting approximately one-fifth of the calories acquired for home consumption. Notably, there is a 93% consumption prevalence of these products among Brazilian children aged 24 to 59 months ^41^ ^,^ ^42^.

HDL-c is known to prevent the oxidation and aggregation of LDL-c in the arteries, thus reducing the risk of cardiovascular disease ^11^. The high prevalence of reduced HDL-c in children appears to be linked to lifestyle factors, particularly inappropriate eating habits ^10^. In our study, a decrease in HDL-c was associated with higher energy and cholesterol intake. A comparable detrimental effect was noted by Souza et al. ^11^, who identified a statistically significant association between candy consumption (equal to or greater than seven times a week) and reduced HDL-c levels in children. This finding underscores the issue of poor dietary choices, as children who consume excessive amounts of candy typically lack a nutritionally balanced diet.

The lack of extensive data regarding levels of physical activity and consumption of food groups, which are primary sources of fats (e.g., oilseeds, animal-derived fats, oils, margarines, fried foods, among others), might have hindered a thorough analysis of the children’s dietary habits. Additionally, the proportion of nutrients relative to the total energy value of the diet was not determined. There was no differentiation as to determine whether the carbohydrates originated from fruits, whole/refined cereals, sugars, or legumes, nor whether the proteins or lipids came from animal or plant origin. Further investigations are necessary to shed light on the relation between nutrient intake, from different food sources, and alterations in serum lipids in the pediatric demographic.

This study was focused on examining the lipid profile of a distinct group in a city located in Central-West Brazil, referencing the epidemiological characteristics and in alignment with the stipulations outlined in the update of the *2017 Brazilian Guideline on Dyslipidemia and Atherosclerosis Prevention* ^1^. As a limitation, it is essential to consider the possible influence of unmeasured variables, such as genetically derived dyslipidemia, biomarkers (like C-reactive protein, apolipoproteins B and AI, fibrinogen, TNF, and IL-6), levels of physical activity, and duration of sedentary time, given that cardiometabolic risk is influenced by a multitude of factors.

In addition, given the nature of this cross-sectional study, which uses baseline data from a randomized clinical trial, it was not feasible to gauge the incidence of dyslipidemia cases or deduce any causal relation based on the findings. For models in the Poisson regression analysis with robust variance adjustment, the outcome variance explained by the independent variables (pseudo R^2^) was low. This may account for a minimal portion of the outcome variability ^29^. Furthermore, the diet was assessed using 24hR (supplemented with direct food weighing in CMEIs), and despite the clear guidelines and comprehensive training provided to the data collection team, there might be instances of underestimation or overestimation regarding the quantity or types of food provided and consumed by the children. Additionally, challenges are posed by the potential memory biases of mothers and/or guardians about the food offered to the children (regarding 24hR), coupled with the absence of data on certain foods and nutrients (like cholesterol, omega-6, omega-3) in Brazilian food composition tables.

A notable strength of this study was its focus on analyzing the lipid profiles of children under 2 years of age. We observed that dyslipidemia research is sparse in the age group from 6 to 42 months. To our acknowledgment, this is the inaugural study conducted in Brazil that probes the association between dyslipidemia and nutrients consumption (encompassing energy, carbohydrates, proteins, fats, and fiber) in children under 24 months. Other commendable attributes of this research include a high participation rate and the direct weighing of food at CMEIs, which is considered the gold standard for evaluating food consumption. This study thoroughly investigated crucial variables in the realm of children’s health, offering estimates and delineating factors associated with lipid profiles. Due to the significant prevalence of dyslipidemia detected in this research, researchers took proactive steps to ensure that all parents or guardians were apprised of the test results. Additionally, they received nutritional advice, and if required, a recommendation for a pediatric consultation.

This study findings underscore the pressing need to devise public health policies aimed at preventing childhood dyslipidemia. These insights should steer further research endeavors, inclusive of extension projects by UFG, to encourage healthy lifestyle habits. These habits encompass weight management, regular physical activity, and nutritional intake tailored to age-specific, caloric, and nutrient requirements, all working in tandem to mitigate the risk of cardiovascular incidents in adulthood. The importance of the dietary guide for children under 2 years old and the protocol for using the dietary guide for children aged 2 to 10 years stands out in this context of health promotion ^43^. CMEIs also hold pivotal importance in fostering nutritional literacy among children and ensuring the quality of meals served, be it in the school lunch or institutional canteen.

In conclusion, a high prevalence of children aged 6 to 42 months exhibited one or more abnormalities in their serum lipid profiles. Notably, the highest observed prevalence rate was for low HDL-c levels. Factors such as an elevated BMI-for-age z-score, older children’s age, and high intake of cholesterol and calories were associated with abnormalities in the lipid profile. Conversely, older age was identified as a protective factor against low HDL-c. Engaging families in offering a balanced, nutritious diet during childhood might very well chart the course toward preempting cardiovascular ailments in this population’s future.
